# Teaching GenerationPMTO, an evidence-based parent intervention, in a university setting using a blended learning strategy

**DOI:** 10.1186/s40814-019-0476-8

**Published:** 2019-07-13

**Authors:** Ana A. Baumann, Melanie M. Domenech Rodríguez, Elizabeth Wieling, J. Rubén Parra-Cardona, Laura A. Rains, Marion S. Forgatch

**Affiliations:** 1 George Warren Brown School of Social Work, Washington University, St. Louis, Campus Box 1196, One Brookings Drive, St. Louis, MO 63130 USA; 2 Utah State University, 2810 Old Main Hill, Logan, UT 84322-2810 USA; 3 Family Social Science, University of Minnesota, 290 McNeal Hall, 1985 Buford Circle, St. Paul, MN 55108 USA; 4 Steve Hicks School of Social Work, University of Texas at Austin, 1925 San Jacinto Blvd 3.130F. STOP D3500, Austin, TX 78712-0358 USA; 5 Implementation Sciences International, Inc., 10 Shelton-McMurphey Boulevard, Eugene, OR 97401 USA

**Keywords:** Pilot study, Blended learning, Therapist training, Parent intervention, GenerationPMTO

## Abstract

**Background:**

Despite the large number of evidence-based practices (EBPs) ready for implementation, they are the exception in usual care, especially for ethnic minority patients, who may not have access to trained health professionals. Providing EBP training as part of a graduate curriculum could help build the pipeline of professionals to provide quality care.

**Methods:**

We conducted a before-after study to determine whether we could implement a blended learning strategy (BL; i.e., in vivo and online training) to teach an EBP in university settings. Feasibility in this pilot was operationalized as knowledge acquisition, satisfaction, fidelity, acceptability, and usability. Using GenerationPMTO as the EBP, our aim was to train graduate students enrolled in Psychology, Social Work, and Family Therapy programs in the EBP in one academic year. Two therapists from a community agency were also students in this pilot. A total of 13 students from five universities were trained in the intervention. Adaptations were made to the intervention and training strategy to optimize training fidelity. Focus groups were conducted with the students to capture their perspective about the training.

**Results:**

Students demonstrated significant knowledge acquisition from baseline (Mean = 61.79, *SD* = 11.18) to training completion (Mean = 85.27, *SD* = 5.08, mean difference = − 23.48, 95% CI = − 29.62, − 17.34). They also reported satisfaction with the BL format, as measured by teaching evaluations at the end of the course. Instructors received acceptable fidelity scores (range of 7–9 in a 9-point scale). Qualitative findings from focus groups showed support for acceptability and usability of BL training.

**Conclusions:**

BL training in university settings can be conducted with fidelity when provided by appropriately trained instructors. BL that integrates EBP and adaptations may be uniquely applicable for training providers in low-resource and ethnically diverse settings. The BL enhanced knowledge of GenerationPMTO was acceptable and usable to students, and was delivered with high instructor fidelity to the training model.

## Background

Despite great progress in developing evidence-based practices (EBPs), the implementation of EBPs remains the exception rather than the rule in usual care settings [1, 2]; the difference in the proportion of people who need these services and those who actually receive them is high [1, 3]. One of the many barriers for providing quality care is the regional variation in the supply of well-trained providers [4]. In addition to a general shortage of providers [5], significant mental health disparities have been documented for racial and ethnic minorities [6–9]. Ample research shows that the number of providers are not appropriately trained to provide culturally competent services; thus, appropriate access to training is needed to successfully address health disparities [5, 10]. Training in EBPs is rarely integrated within Psychology, Social Work, and Family Therapy curricula [11–13], despite the fact that the Institute of Medicine has identified EBP skill as a core competency for twenty-first century health professionals [14, 15] and that scholars have identified training in EBP as a scientific, ethical, and financial solution to the problem of improving the quality of care [11]. No other area of health care would tolerate such low level of quality control [16].

There is consensus on the importance of training students from Psychology, Social Work, and Family Therapy on EBP packages to increase practice effectiveness and prepare a competent workforce [16–20]. The advantages of training students in EBP during their graduate work are twofold: (a) from a clinical perspective, to improve service delivery into the future; and (b) from a research perspective, to promote students’ use of research to test theoretical underpinnings, practices, and to evaluate the EBP’s mediators and moderators [18]. Early exposure to EBP training can strengthen students’ present and future practice as well as their scholarly work while benefiting the patients and communities that they serve [21]. Even if training in EBP is established in university settings, however, the shortage of supervisors qualified to train providers, and the small amount of available time to provide the supervision required for effective service delivery are challenges that need to be addressed [16, 20]. Practitioner specialty training is typically provided via didactic workshops. While didactic workshops increase knowledge, they often do not change provider behavior [22, 23]. Moreover, training workshops are expensive and costs of ongoing training and consultation remains a barrier to training access [19].

Blended learning (BL), the combination of traditional face-to-face and online instruction, is a promising strategy for training therapists in university settings and can serve as a bridge between students and well-trained EBP practitioners from different institutions. The use of BL learning is increasing in higher education settings around the world [24], and it has been predicted that it will be the “new normal” in higher education [25] as research suggest comparable outcomes across in vivo and BL modalities in achieving knowledge transfer [26]. BL training can be a promising means of scaling up an EBP in different settings [22, 27] and can be a promising approach to address the challenges of training future providers; particularly culturally competent providers delivering care for minority population.

### Study purpose

This pilot study aimed to test the feasibility of using a BL training strategy to train EBP across five university settings, where feasibility was operationalized as knowledge acquisition, satisfaction, fidelity, acceptability, and usability. We employed GenerationPMTO® in this course as an example of EBP. GenerationPMTO is an evidence-based parenting intervention that has demonstrated effectiveness with clinical and prevention samples. Randomized controlled trials have found increased positive parenting practices, decreased coercive parenting to serve as mediators for improved clinical outcomes, including decreased delinquency, youth arrests, internalizing problems, and deviant peer association, up to 9 years after intervention [28–30]. GenerationPMTO training has been documented as having sustained effects for interventions provided by community practitioners in community mental health and child welfare systems in the USA and internationally [31]. The GenerationPMTO team is committed to improving access to care while adhering to the most rigorous standards of fidelity. Competent delivery is assessed via video recordings and rated with a fidelity measurement tool, the Fidelity of Implementation Rating System (FIMP; [32]), which has shown to have predictive validity for pre/post changes in observed parenting practices and parent child outcomes [28, 33–35].

Our team works with populations all over the world. Particularly relevant to this study is our work with ethnic minority populations and the motivation to address health disparities by training providers likely to serve vulnerable populations. From a GenerationPMTO perspective, taking culture into account in treatment serves to maintain fidelity [32]. Scholars within the GenerationPMTO team have focused on addressing health disparities nationally and internationally, and have become experts in adaptations of EBPs as well as experts in GenerationPMTO. Because of our collective experience with scale up of the intervention [36–39], we explored an innovative platform to improve the capacity to serve ethnic/racial minority patients. We believed that the BL strategy could serve as a model for training across EBPs in general, and for those programs seeking to train practitioners to address health disparities in particular.

A secondary aim of this study was to examine the fidelity of the training. Much has been written about the fidelity of therapists when delivering interventions and the importance of this construct as mediator for parent outcomes [33, 40, 41] but not a lot is known about the fidelity of the training at the provider level. To be able to train therapists in university settings, we needed to change both the mode (from in person to blended) and the frequency (18 months to one academic year; see “Method” section). As such, our team examined whether the fidelity of the training would be sustained in this different context.

## Method

### Sites and participants

Participants were 14 students in five university sites in five states: Florida, Michigan, Minnesota, Missouri, and Utah. One student from Missouri dropped out after the first semester due to personal conflicts. Each group of students from the five sites worked with the following families: US White American families, Latinx families, Karen refugees, divorced families, and US born families. The instructors and coaches were Latinx (two Brazilians, one Puerto Rican, and one Mexican). All instructors participated in the study: two led the course (AB and MDR), in addition to providing local coaching to their site students, and two provided coaching to their students (LW and RPC). The GenerationPMTO developer and her team (LR and MF) closely followed the adaptation to the training and provided mentoring during the study process. The institutional review boards at all universities approved this research and students were individually consented by the first author.

### Design

#### The intervention

GenerationPMTO follows a training model, with a certification process that entails continued monitoring of fidelity and outcomes based on direct observation and coaching of intervention sessions [31, 34, 42]. Certification at the level of therapist involves attending in vivo workshops, conducting clinical work, and incorporating coaching feedback until a therapist achieves fidelity certification scores. GenerationPMTO training to certification requires approximately 12 to 18 months [36], although training approaches have varied across implementation sites.

Traditionally, GenerationPMTO training has been conducted in community service agencies with practicing professionals. Training in an academic setting with graduate students presented a number of challenges. First, we had to change the dose and frequency of training events to map onto academic semesters. We did so by sharing proposed agendas with topics of trainings with the treatment developer and her team (LR and MF). Second, as part of the training process, students were required to practice their skills by conducting parenting groups. The standard GenerationPMTO group intervention was 14 sessions, which was not feasible for our purposes. The treatment developers adapted the group manual for ten sessions. Finally, our goal was to test the feasibility of the BL approach for training students in EBP by simply examining if we could conduct a BL course over an academic semester.

#### The BL course

The course was led by the two first authors, who are GenerationPMTO certified as Specialists, Coaches, Trainers, and Fidelity Raters. Students and trainers met for 90 min weekly over two academic semesters, for a total of 26 weeks. All sessions were recorded for fidelity checks. We used synchronous (live) online meetings for class and coaching using the Google Hangouts platform and asynchronous discussion boards for ongoing communication among students, teachers, and mentors. Asynchronous materials (e.g., treatment manual, readings) were kept in a DropBox folder. Training materials included directed readings, relevant videos, PowerPoint slides, and other course documents (e.g., blinded transcripts of GenerationPMTO sessions).

In the first semester, students were expected to read scientific articles and submit discussion questions. To practice GenerationPMTO skills, students conducted fictional parenting groups, leading sessions with friends or students. In the second semester, trainers emphasized skills practice over readings. Students conducted parenting groups with parents from their community; coaching was provided in class. Students uploaded their videos to a HIPAA-compliant portal. In four of the five sites, students had access to a GenerationPMTO-certified specialist who provided onsite coaching as needed; the fifth site received monthly online coaching with the first author.

#### Implementation strategies

Proctor and colleagues [43] have urged precision in defining and operationalizing strategies to adopt and integrate health innovations into care [44]. Following their recommendations, Table 1 describes the components of our study in which GenerationPMTO certified trainers (the actors) trained (action) doctoral students (action targets) in GenerationPMTO during two academic semesters (temporality) with one encounter of 90 min per week plus homework assignments. Practice required students to conduct parenting groups.

**Table 1:** Components of the GenerationPMTO training at university settings

Strategy	Literature definition/Our specification	Justification	Target	Outcomes/Dose
Educational				
Active educational meetings	*Hold meetings targeted toward students*/*providers to teach them about the clinical innovation* Online classes: one encounter of 90 min per week for two academic semesters. Classes entailed both discussions of theory as well as role plays to strengthen skills	Theoretical [61], knowledge, self-efficacy [62] Empirical [22]	Students’ knowledge	Implementation outcomes: Knowledge gain Model fidelity (instructors as well as students) Dose: three knowledge surveys: October and November of 2015 and May of 2016
Train-the-trainer	*Master trainers teach the content of the EBP as well as the principles of active learning strategies to supervisors who then train their therapists on EBPs utilizing empirically-supported training methods* Close monitoring of the teaching technique by the treatment developer and GenerationPMTO mentors to guide AB and MDR teaching in class. Teaching of students by certified coaches (AB, MDR, JRPC, EW)	Theoretical: adaptation of the GenerationPMTO implementation model to an academic setting [53, 63] Empirical [64]	Students’ knowledge and skills building	
Education through peers	*Develop group of providers that will implement the EBP and develop ways to learn from one another to foster implementation* [44] Students provided feedback on each other’s performance during sessions and role plays in class using the FIMP structure	Theoretical: knowledge [62]	Students’ knowledge and skills building and peer-to-peer support	
Quality management				
Develop and organize quality monitoring system	*Develop and organize systems and procedures that monitor clinical processes and*/*or outcomes for the purpose of quality assurance and improvement* Fidelity ratings as performance feedback to students and coaches; share notes and gather feedback on class preparation with treatment developer and team; mid-semester and end of semester qualitative assessment of course with coaches and students; implement student and coach goal-settings for skill acquisition in GenerationPMTO	Theoretical: reflecting and evaluating; available resources; access to knowledge [62] Empirical [65]	Lead trainers’ fidelity to the model while adapting the training Students’ knowledge and skills building	Implementation outcomes: Fidelity to the model Dose: weekly coaching during class for students; one meeting per semester with lead trainers and treatment developer
Audit and feedback	*Collect and summarize clinical performance over a specified time period and give it to clinicians to monitor*, *evaluate and modify behavior* Verbal and written qualitative feedback were provided to students and coaches on their GenerationPMTO knowledge and skills performance	Theoretical: knowledge, peer pressure [62] Empirical [66, 67]		
Consultation	*A process of interaction between two professionals*- *the consultant*, *who is a specialist*, *and the consultee*, *who invokes the consultant*’*s help in a work problem that he believes is within the consultant*’*s area of specialized competence* Small samples (10–20 min) of videos from their sessions with parents were observed in class and students received feedback on their GenerationPMTO skills	Theoretical: knowledge, self-efficacy [62] Empirical [23]		

#### Data gathering approach

Quantitative and qualitative data were collected simultaneously, giving equal weight to both types of data (QUAN + QUAL) [45], using the qualitative data to complement the quantitative data to provide deeper understanding of the students’ perspective. We used quantitative measures to examine students’ knowledge gain, the acceptability and usability of the BL platform, and to evaluate the teaching process and the fidelity of the teaching process. The qualitative phase aimed to explore the students’ experiences with the course. The two processes were used to provide significant enhancement of the results [46].

## Measures

We used a pre-post study design with follow-up. Feasibility in this pilot was operationalized as knowledge acquisition, satisfaction, fidelity (at the instructor level), acceptability, and usability. Thus, quantitative measures included a knowledge survey, a teaching evaluation, and measures of acceptability and usability. Additionally, we conducted a semi-structured group interview.

### Knowledge survey

The survey comprised 17 open-ended questions about core GenerationPMTO components. Answers were rated using a 9-point scale that ranged from 1 (*no evidence of competence*) to 9 (*exemplary*). All answers were coded by the first two authors; disagreements were resolved by consensus. Scores were calculated as a percentage of the total.

### Acceptability and usability of technology survey

An adapted version of the Hsieh et al. survey was administered in December 2015 and May 2016 [47]. The survey asked about the three platforms used: Google Hangouts, Dropbox, and the ISII portal. Sample questions were “the BL approach fits into the way I like to take courses”; “I think other classes should take a BL approach”. Items were rated on a Likert-type scale ranging from 0 (*strongly disagree*) to 100 (*strongly agree*); higher scores indicated high acceptability and usability of the platform.

### Teaching evaluation

At the end of the course, students answered a 21-item survey evaluating instructors’ knowledge, engagement, assignments, and general course observations. Sample questions were “Instructors were knowledgeable about the subject”; “I learned GenerationPMTO skills in this course”. Items were rated on a 10-point Likert-type scale.

### Qualitative group interviews

Participants were interviewed in a group to explore their experiences and perceptions regarding the acceptability and feasibility of the course [48]. Group interviews were conducted with each site independently and led by the third author after the Fall 2015 and by the fourth author after the Spring 2016 via online video interviews. Each group was audio recorded and then transcribed for purposes of analysis.

The interview protocol started with a review of the study guide, general description of the interview process, and assurance of individual participant and group confidentiality. Participants had the option to be interviewed separately. Conversations began with an open-ended grand tour question: “What can you tell us about your overall experience as a participant in this BL training?” Follow-up questions explored BL training components, (e.g., “How was it for you and your team to be trained online?”), coaching (e.g., “How did you experience the coaching as part of the overall training model?”), and content (e.g., “How did you perceive your growth – knowledge and skills – related to GenerationPMTO after participating in this BL training?”).

### Fidelity rating of the instructors

GenerationPMTO has a well-established fidelity measure, the Fidelity of Implementation Rating System (FIMP, [32]). FIMP evaluates competent adherence to the intervention on five dimensions: Knowledge (understanding of model, principles, and strategies), Structure (session management, responsiveness, sensitive pacing), Teaching (balancing verbal/active instruction to promote mastery), Process Skills (use of clinical skills to create supportive learning environment), and Overall Development (family engagement, growth, satisfaction). Each dimension is rated on a 9-point Likert-type scale, in which 1–3 reflects *needs work*, 4–6 *acceptable work*, and 7–9 *good work* [44]. Certified fidelity raters score each dimension independently and the session receives a mean score of all dimensions. FIMP has shown predictive validity in observed parenting practices [34, 49, 50], child outcomes [35], and treatment outcomes [34, 35, 49, 50]. The measure has been adapted to assess workshop and training activities. For this study, a total of 14 segments of 20 min each from classes in 2015 and 2016 were selected for reliability testing. The segments were selected with the goal of providing a range of topics (e.g., introducing encouragement; troubleshooting discipline; providing coaching to students) to allow for a range of scores and participation of co-leaders in the segments. Fidelity rating of random 20-min segments is standard practice in GenerationPMTO to assess model adherence and competent delivery at the therapist level [45]. For reliability purposes in this study, two segments from 2015 (segments 4 and 6) and one segment from 2016 (segment 5) were checked by two additional certified fidelity raters not involved with this study; the scoring of the videos was coordinated by LR. The fidelity score of the instructors was obtained by averaging the scores on each component to get an overall score per each segment for each instructor.

#### Sample size

Students were doctoral candidates (*n* = 8), master students (*n* = 1), postdoctoral (*n* = 1), assistant professor (*n* = 1), and community practitioners (*n* = 2). Students were 11 women and two men between 24 and 34 years of age (Mean = 28.25; standard deviation = 3.5). Participants were recruited because they were students from three faculty that have been collaborating for a number of years in a programmatic line of research adapting GenerationPMTO for immigrant families in the USA and internationally. In one site (WU), the community therapists from an agency were enrolled in the course as part of a collaboration between the first author and the agency to train their therapists in GenerationPMTO.

#### Data analyses

Quantitative data were analyzed by calculating frequencies and descriptives. *T* tests and repeated measures analysis of variance were used to test significance of mean differences.

A thematic analysis approach [51] was used to analyze open-ended interview data. After each set of interviews (fall and spring), the key interviewers (EW and RPC) transcribed the data and conducted a first level inductive analysis of each group. A listing of emerging themes was developed that incorporated relevant quotes and a brief summary statement for each group. This within-group analysis was sent to each participant member across university settings for member checking. Group participants were asked to verify accuracy and review information, and to further deidentify the thematic analysis before the document was shared with BL primary trainers. After participants verified and approved the thematic analysis, a second level of analysis was conducted to integrate overlapping themes across groups. A third level of analysis occurred using the across-group thematic analyses from fall and spring semesters. Key overlapping themes related to acceptability of the BL model are presented below.

## Results

### Quantitative

#### Knowledge

Students’ scores showed improvement over time. October 2015 mean Knowledge scores reflected students’ emerging knowledge of GenerationPMTO interventions (Mean = 61.79, *SD* = 11.18). December 2015 Knowledge scores showed improvement (Mean = 77.73, *SD* = 5.14). In May 2016, more improvement was evident (Mean = 85.27, *SD* = 5.08). A repeated measures within groups (time) ANOVA showed statistically significant differences, *F* (2, 24) = 45.01, *p* < .001, η_p_^2^ = .790. A small partial eta square (η_p_^2^). is .01, a medium one is .06, and a large one is .14. Mauchly’s test of sphericity indicated the assumption of sphericity was observed, χ^2^(2) = .875, *p* = .646. Paired samples *t* tests showed statistically significant differences between October 2015 and December 2015, *t*(12) = − 6.34, *p* < .001, mean difference = − 15.94, 95% CI (− 21.41, − 10.46), October 2015 and May 2016, *t*(12) = − 8.33, *p* < .001, mean difference = − 23.48, 95% CI (− 29.62, − 17.34), and December 2015 and May 2016, *t*(12) = − 3.41, *p* = .005, mean difference = − 7.54, 95% CI (− 12.36, − 2.72). Our findings reflect statistical significance (indicated by the *p* value of less than .001 and confidence intervals non-overlapping with 0) and clinical significance (indicated by the large size of the partial eta squared statistic, η_p_^2^). Regardless of these strong findings, the sample is small and results should be considered with caution.

#### Usability of technology

Students were asked to provide ratings on four items in Fall 2015 and Spring 2016. Ratings for the BL model were similar in December 2015 (Mean = 67.06, *SD* = 17.80) and in May 2016 (*Mean* = 67.89, *SD* = 16.90). Usability of DropBox was rated in the fall (Mean = 73.25, *SD* = 17.98) and the spring (Mean = 74.88, *SD* = 16.30). The usability of the ISII portal received the lowest rating, with similar ratings in the fall (Mean = 51.69, *SD* = 27.02) and spring (Mean = 51.35, *SD* = 29.46). The one area with a shift in students’ ratings was with Google Hangouts, which was higher in the fall (Mean = 73.25, *SD* = 26.15) than the spring (Mean = 63.32, *SD* = 24.28).

#### Teaching evaluation

Overall, students reported a positive BL experience (see Table 2). Students perceived instructors to be knowledgeable about the topic, enthusiastic, and responsive to the students. There was mixed feedback regarding the assignments, where some students reported less enthusiasm with the assignments than others. Despite challenges and limitations, students reported significant learning of GenerationPMTO skills and strategies.

**Table 2:** Teaching evaluation (*n* = 14)

Teaching evaluation	Mean (SD)
Instructors were knowledgeable about the subject	1.09 (0.30)
Instructors were prepared for the class	2.09 (1.22)
Instructors presented materials in a way that helped me learn	1.81 (0.75)
Instructors encouraged participation	1.09 (0.30)
Instructor answered student’s questions	1.18 (0.30)
Instructors were enthusiastic about teaching	1.09 (0.30)
The pace of the course was just right	2.09 (0.70)
I would recommend these instructors to others	1.27 (0.65)
Instructors had set agendas that facilitated the learning process	1.63 (0.50)
Instructors used active teaching	1.09 (0.30)
Instructors used good questioning process	1.09 (0.30)
Assignments	
The readings/homework assignments were at the right level of difficulty for the course	1.63 (0.50)
Assignments given for class interested me	2.18 (1.17)
Assignments were about the right length	2.45 (1.04)
General course	
I learned GenerationPMTO skills in this course	1.18 (0.40)
This course improved my GenerationPMTO knowledge	1.18 (0.40)
I learned from my peers	1.54 (0.69)
Overall, the quality of the course was good	1.09 (0.30)
If offered again, I would recommend this course to others	1.18 (0.40)

### Qualitative findings

Qualitative findings illustrate a shared sense of satisfaction from having the unique opportunity to (a) receive EBP training, (b) participate in an innovative BL training approach, and (c) network with key parenting researchers and educators in the field. Students also reported important challenges, which primarily centered on (a) technological difficulties, (b) transporting practice in the model to real-world settings, (c) balancing fidelity to the model while remaining responsive to contextual and cultural issues that were salient in the lives of both students and the parents that they served, and (d) lack of a clear plan to continue training toward model certification within the university setting.

#### Acceptability

One of the most relevant qualitative themes across groups was the students’ appreciation for the connection across sites. This was particularly salient because there are few opportunities for individual practitioners to learn GenerationPMTO, as it is typically implemented throughout systems of care in the US and abroad. Related to connection, exposure to diverse settings, perspectives, and experiences associated with the various sites was highly valuable to students. One participant stated: “We had to learn to connect across 5 different sites and time zones…and make it feel like a classroom. That was masterful.” Another student mentioned appreciation for the engagement process, “I really appreciated the effort to develop and use this blended model with us, to staying engaged, and to working through the frustrations with technology.”

In general, students shared that the BL experience was valuable and that the quality of learning exceeded their expectations, as one student affirmed:

Overall, despite the small problems we ran into, I had a great experience. I was surprised to find out how much I learned when we applied it outside of class, and it was a nice reinforcement to see how much I had learned.

The online component of the training was considered a positive aspect of the learning experience. Despite challenges associated with technology, students reported that this component made it possible to bring down “distance barriers” and expose participants to unique opportunities to learn from fellow instructors, as well as GenerationPMTO trainers and researchers across the world. One student reflected on these issues:

Online component afforded us the opportunity to work full time and be able to take this training – connecting remotely made it all possible…and to use technology to access other parts of the course materials and submit our videos was perfect for us.

In addition to breaking down distance barriers, the online component of the training made the materials available, which was essential to allow students to meet their training goals while balancing other responsibilities. Another student reflected:

Another bonus and opportunity of this model is that if we need to miss a session, we can access it easily because the recordings are posted…this way we do not miss Ana and Melanie but also stay connected to what the other groups are saying.

The structure of the course was considered acceptable. Students reported that the course was well planned and that co-facilitators quickly adapted to the online technology. One student said, “Everyone was very engaged. We had incredible guests…”, “Ana and Melanie were always attentive to respond to different needs and goals of the group.” Students uniformly highlighted the strong collaboration that they observed between the lead trainers, as one student said, “You could tell how well Ana and Melanie worked with each other…It was like a dance…they supported each other and complemented each other very effectively.” Finally, students reflected about the importance of team cohesion and collaboration that they experienced in each of the sites, as one student affirmed: “The team was incredibly cohesive and collaborative throughout the training…it was very unique.” Another student expressed a similar reaction, “Being on a team was critical…there were so many logistics involved. I appreciated the way the team worked together. I would not have done it otherwise.”

#### Knowledge gained

Participants were surprised by the amount and depth of learning they accomplished, which they attributed to the quality of instruction by the lead trainers, support received by site coaches, and various components of the BL course (e.g., online components, site practice, guest speakers). According to students, the integration of these experiences resulted in a solid learning experience. One student reflected on her overall learning, "Tremendous increase in GenerationPMTO^1^ knowledge and skills, with an emphasis on growth in process skills – the role play practices and fictional sessions helped to integrate and solidify knowledge." Interestingly, students indicated that the learning process extended beyond the intervention and work with parents, as one participant expressed, “I started to make the connection between GenerationPMTO and so many of the other works I read about and apply in the field...it was eye opening.” Students connected their knowledge with other settings, as one person mentioned: “GenerationPMTO is highly applicable and useful across relational settings, even if not working with parents.”

#### Completing the cycle with real-world application

Students uniformly expressed that the real-world application of the training was essential to solidify their training. Without this, students reported, they could not have fully learned the intervention, which included developing the skills necessary to adapt to contextual challenges in real world settings. One student described the value of training that included running groups in community-based settings, “What was most helpful, by far, was leading a group and then getting coaching on it. I feel I improved as a GenerationPMTO leader a lot through this experience.”

A relevant theme reported by participants was the critical role played by site coaches when transitioning to real world settings. Students reported that this support enabled them to achieve many goals essential to running groups, such as obtaining funding to implement groups, managing politics with community sites, addressing challenges presented in groups, and ensuring a fit between the model and the contextual and cultural realities of clients. As one student expressed, “It was wonderful to have a direct access to our local coach as whenever we faced a challenge, we would reach out to her knowing that we would have a fast response either via email, phone, or face-to-face.”

#### Challenges

##### Technology

The most common challenge reported involves technological issues. For example, during class sessions, sites not actively participating were asked to mute their microphones to decrease interference. Muting microphones presented another challenge: as one student affirmed, “When audio was muted it was easy to disengage at times…it felt stressful because I didn’t want to look like I wasn’t paying attention.” Additionally, students were asked to upload their videos to the ISII portal for coaching which was under construction at the time. One student affirmed: “Technology became a barrier at times - video uploads were very frustrating because it would take so long. I could not do anything else on my computer when I was uploading videos.”

##### Workload expectations

Participants reported that workload expectations specified at the start of training did not correspond to the actual workload that was required. Key workload activities included time to complete course readings, recruitment of participants for the applied component, planning and implementing groups, and engaging in coaching. Students also reported that whereas fictional groups were highly relevant, they were time consuming. Interestingly, students recommended an increase in coaching during the fictional phase of training to solidify skills. One student summarized, “The only recommendation for the future would be to increase coaching of fictional sessions throughout the training so that team can use the feedback to build their skills from one week to the next.”

##### Balancing fidelity and fit

As students reflected on their future careers and continuing mastery of GenerationPMTO when serving diverse populations, trainees reflected on balancing model fidelity with flexibility in order to adapt to key contextual and cultural issues. This theme was particularly pronounced among students who worked with parents exposed to substantial contextual challenges such as discrimination and poverty. One student appreciated having direct access to her onsite coach, who has developed a line of research focused on cultural adaptation:

Because our site supervisor specializes in cultural adaptation, there was a feeling of comfort to know I had direct access to a person with this expertise…Someone who has studied this, ran groups in Spanish, and managed many of the contextual and cultural challenges we experienced when we ran our group.

#### Integration of mixed methods

We found convergence across methods. As shown in Table 3, both quantitative and qualitative results support the acceptability and usability of the BL training. For example, both sets of results show increase in knowledge by the students. Both indicate that the BL platform was acceptable, although students highly recommend changes in the technology for a future study.

**Table 3:** Integration of mixed method results demonstrating convergence of findings

Approach	Quantitative	Qualitative
Question	Is the BL a feasible implementation strategy to train EBP?	
Answer	Yes: Students reported that BL was feasible	Yes: Students articulated that the mix of online and in vivo components of the class were feasible and helped them acquire GenerationPMTO knowledge
		No: participants complained about issues with technology, too many platforms and issues uploading videos for supervision
Question	Is BL an acceptable training?	
Answer	Yes: Students had a significant increase in GenerationPMTO knowledge	Yes: BL training was acceptable and students were thankful for the opportunity of meeting GenerationPMTO mentors

##### Fidelity rating of the instructors

**Fig. 1:**
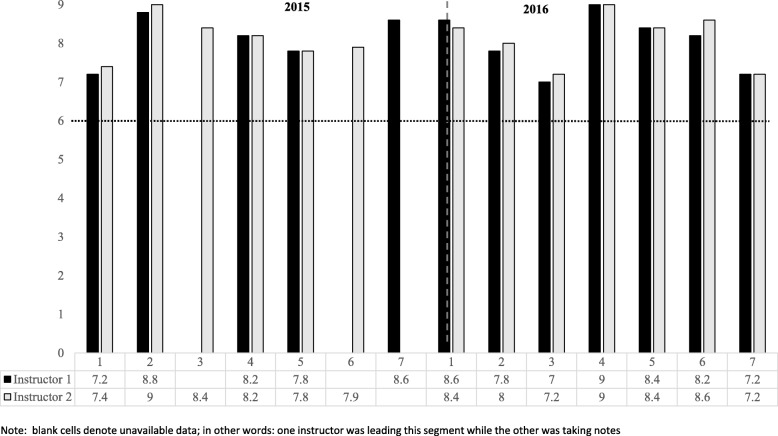
Fidelity rating of instructors 1 and 2 in selected segments during the 1-year training. A score of 6 or higher reflects adherence to the model

Figure 1 shows the scores of each instructor in each of the segments. The scores for both instructors in all sessions were in the range of 7–9, reflecting good to excellent work. The segments in 2015 had higher variability in scores: some segments did not have scores for both instructors because one instructor led throughout; thus, there was no active co-leading. This was reflected in fidelity raters’ comments to instructors: “Was agenda clear to both co-leaders? Difficult communication about task at hand – setting up goals or creating ground rules?” The segments in 2016 show growth in collaboration among co-leaders, reflected in one rater’s comments: “There is super understanding of content being delivered. Co-leaders as a team make sure students are on board.”

## Discussion

This pilot study aimed to evaluate the feasibility use of a BL platform to train an EBP (GenerationPMTO) in five university settings over the course of one academic year; with feasibility operationalized as knowledge acquisition, satisfaction, fidelity, acceptability, and usability. Findings show that the BL platform was accessible, had utility, and increased students’ knowledge about GenerationPMTO content and processes. We were able to simplify the training to fit the university setting, i.e., classes were conducted by certified coaches, videotaped, monitored for fidelity, and designed so that the intervention developer and her implementation team were able to “attend” and interact with students from different sites simultaneously. Most importantly, instructor training fidelity was high indicating that BL can be a useful platform to train therapists without affecting the fidelity to the training.

Important strengths in collaboration between program developer and course trainers made this pilot study possible. The trainers (AB and MDR) had a long history of collaboration with key stakeholders across sites (EW and RPC) which minimized the challenges inherent in conducting a five-campus collaboration. In addition, the GenerationPMTO model includes specific training on co-leaders collaborating. It is possible that replications conducted with other treatment packages or by trainers with limited experience collaborating may be less successful. Furthermore, all of the authors have significant experience in training university students.

Over the course of training, we had significant challenges with technology. Results show a drop in the usability of Google Hangouts from Fall of 2015 to Spring of 2016. At the beginning of Spring semester, Google Hangouts updated its software causing several connection problems. In moving forward, we need to weigh the advantages and disadvantages of using publicly available platforms. Preparing for possible disruptions on free platforms can be accommodated by adding class time or by switching to asynchronous method of communication when needed. Because of the ubiquitous nature of online learning, many university campuses have advanced distance learning tools. Since this study, the second author has become proficient in the use of Canvas for online teaching. Using such a platform would address many discontinuities and provide protection of sensitive data (e.g., video uploads), eliminating the need for students to access many different platforms.

Workload for this course was a notable challenge. When our pilot training program started, we were still negotiating how and when students should practice skills with parenting groups. Challenges included recruitment of parents and finding facilities to host the groups. Some students had to recruit parents for groups in the community, which would not be an issue with training at an agency. Groups required cost sharing from sites (e.g., churches) and local coaches to address expenses associated with location rent, dinners, on-site childcare, and transportation support for participants. Projects in underserved communities faced the need to gather necessary resources to ensure the parents’ engagement and retention in the intervention. Our course focused on reducing health disparities and so we required students to work with marginalized people. This may have been too stringent a criterion to require for training. Additionally, universities often have in-house training clinics. Establishing a long-term relationship with the universities where the BL course is offered could eventually result in a stable population for training purposes (e.g., establishing an ongoing practicum site focused on offering parent-training). Collaboration with other programs on campus could result in mutually beneficial relationships (e.g., Early Childhood Education students may need to practice skills that could be rehearsed in a child care situation thus providing free child care for parents attending the groups).

The group of trainees was very small and heterogeneous. The students were part of our centers and were contributing to our overarching line of research to scale up GenerationPMTO to ethnically diverse populations in the USA and internationally [37, 38, 41, 52–54]. In spite of having been exposed to some preliminary GenerationPMTO before, statistically significant gains in knowledge by the students were evident after focused training. Studies have found the strength of blended learning in training therapists with large samples (e.g., [55–57]) and we now see that it is also useful with smaller heterogeneous groups in academia. Finally, research has shown that online training can have similar effect in terms of improving provider knowledge compared to in vivo training [57], but this research has not been extended to document improvement in the acquisition of skills. Our study is unique in indicating skills gains based on students’ self-reports in our focus groups; however, we should have caution with the small sample size. In future evaluations, we would suggest that students’ clinical sessions are rated using the FIMP rating system to verify the gain in skills. We predict that the training’s unique use of behavioral rehearsal and coaching would account for gains in clinical skills. Our experience in training therapists suggests, however, that we would need for at least three training groups for the therapists to feel comfortable and start showing adherence to the model [58].

This project involved a group training, requiring a minimum of two therapists for a group of 6–8 parents. GenerationPMTO has also developed training for individual therapists. We opted to do a group training because our group aimed to capitalize on the social component that is important for minority population [59, 60]. GenerationPMTO training is tailored to the agencies where it will be delivered with a more cursory review of theory and research relative to applied skills development. In our case, the academic context called for a deeper dive into theory and research findings. Given the time allotted (two semesters), focusing on group delivery seemed more feasible. It also was an excellent fit to the context of our collective work, which has been delivering group interventions. The knowledge acquired by students could certainly be applied at the individual level. To deliver individual level therapy, however, student would need more supervision on the part of the trainer which was not feasible for us to accommodate in this pilot project.

An important result of this evaluation of the BL model was the clear enthusiasm that this training generated for our university students. Starting in the Fall 2019, the second author will begin to teach a standing course at her university in the GenerationPMTO model that will follow the BL course approach. All the materials will be uploaded to Canvas, the university’s learning management system, to address the earlier technological limitations. The department has also acquired a Zoom account, allowing for more stable form of communication with invited speakers.

While the training within university settings will not result in official certification in the GenerationPMTO model, it does allow for the development of skills in delivering this EBP that may be carried out regardless of the setting that clinicians are in. We have described early approaches to GenerationPMTO certification [53]. As the intervention is being scaled out to a variety of settings, technology has been incorporated in the training. Specifically, practitioners are being trained to implement GenerationPMTO over the phone in British Columbia and most recently, an in person training with a blended online-video approach is taking place. We are closely following the fidelity to the model and examining outcomes as we adapt the trainings to increase the reach of our intervention.

Overall, the BL strategy shows promise in training EBPs. This initial study aims to support a long-term commitment from our team to further equip a scientific community to address health disparities through collaborative teams committed to innovation and dissemination of knowledge and skills. Using BL models as a viable training strategy helps to address this gap in academic settings and increases the potential for EBPs to be further implemented and adapted for various cultural and population needs. Adopting BL models in university settings has noteworthy implications for broader dissemination to low-resource settings and communities throughout the world.

## Abbreviations

BL: Blended learning
EBP: Evidence-based practice
*N*: Number
*SD*: Standard deviation

## Footnotes

1 When this study was conducted, the name of the intervention was Parent Management Training, Oregon model (PMTO). The name of the intervention has since been changed to GenerationPMTO.

## References

[1] Kazdin AE (2017). Addressing the treatment gap: a key challenge for extending evidence-based psychosocial interventions. Behav Res Ther.

[2] U.S. Department of Health and Human Services, Administration for Children and Families. Child maltreatment 2010 [Internet]. 2010 [cited 2018 Nov 2]. Available from: https://www.acf.hhs.gov/archive/cb/resource/child-maltreatment-2010.

[3] Kamal R, Cox C, Rousseau D (2017). For the Kaiser Family Foundation. Costs and outcomes of mental health and substance use disorders in the USCosts and outcomes of mental health and substance use disorders in the USJAMA infographic. JAMA..

[4] Knickman J, Krishnan R, Pincus H (2016). Improving access to effective care for people with mental health and substance use disorders. JAMA..

[5] Hoge M, Morris J, Daniels A, Stuart G, Huey L, Adams N (2007). An action plan for behavioral health workforce development.

[6] Alegria M, Atkins M, Farmer E, Slaton E, Stelk W (2010). One size does not fit all: taking diversity, culture and context seriously. Adm Policy Ment Health Ment Health Serv Res.

[7] Nelson A (2002). Unequal treatment: confronting racial and ethnic disparities in health care. J Natl Med Assoc.

[8] U. S. Department of Health and Human Services, Centers for Disease Control and Prevention, National Center for Health Statistics (2015). Health, United States, 2014. Washington, DC: Government Printing Office. Available online: https://www.cdc.gov/nchs/data/hus/hus14.pdf.

[9] Creedon TB, Cook BL (2016). Access to mental health care increased but not for substance use, while disparities remain. Health Aff (Millwood).

[10] Kazdin AE, Blase SL (2011). Rebooting psychotherapy research and practice to reduce the burden of mental illness. Perspect Psychol Sci.

[11] Rubin A (2011). Teaching EBP in social work: retrospective and prospective. J Soc Work.

[12] American Psychological Association (2006). Evidence-based practice in psychology. Am Psychol.

[13] Crits-Christoph P, Frank E, Chambless DL, Brody C, Karp JF (1995). Training in empirically validated treatments: what are clinical psychology students learning?. Prof Psychol Res Pract.

[14] Greiner AC, Knebel E, Institute of Medicine (US) Committee on the Health Professions Education Summit (2003). Health professions education: a bridge to quality [Internet].

[15] O’Connell ME, Boat T, Warner KE (2009). Preventing mental, emotional, and behavioral disorders among young people: progress and possibilities. Vol. 7.

[16] Insel T. Quality counts. [Blog Post]. The National Institute of Mental Health Information Resource Center. 2014. Available from: https://www.nimh.nih.gov/about/directors/thomas-insel/blog/2015/quality-counts.shtml.

[17] Beck JG, Castonguay LG, Chronis-Tuscano A, Klonsky ED, McGinn LK, Youngstrom EA (2014). Principles for training in evidence-based psychology: recommendations for the graduate curricula in clinical psychology. Clin Psychol Sci Pract.

[18] Howard MO, Allen-Meares P, Ruffolo MC (2007). Teaching evidence-based practice: strategic and pedagogical recommendations for schools of social work. Res Soc Work Pract.

[19] Cook JM, Schnurr PP, Biyanova T, Coyne JC (2009). Apples don’t fall far from the tree: influences on psychotherapists’ adoption and sustained use of new therapies. Psychiatr Serv.

[20] Lack CW, Doan R (2018). Training in evidence-based psychological practice at the master’s level. J Cogn Psychother.

[21] Melnyk BM (2014). Speeding the translation of research into evidence-based practice and conducting projects that impact healthcare quality, patient outcomes and costs: the “so what” outcome factors. Worldviews Evid-Based Nurs.

[22] Beidas RS, Edmunds JM, Marcus SC, Kendall PC (2012). Training and consultation to promote implementation of an empirically supported treatment: a randomized trial. Psychiatr Serv.

[23] Beidas RS, Cross W, Dorsey S (2014). Show me, Don’t tell me: behavioral rehearsal as a training and analogue Fidelity tool. Cogn Behav Pract.

[24] Graham CR, Woodfield W, Harrison JB (2013). A framework for institutional adoption and implementation of blended learning in higher education. Internet High Educ.

[25] Norberg A, Dziuban CD, Moskal PD (2011). A time-based blended learning model. Horiz..

[26] Pereira AS, Wahi MM. Comparison of didactic, technical, role modeling, and ethics learning acquisition in undergraduate online versus face-to-face modalities. J High Educ Theory Pract. 2018;18(5) Available from: https://articlegateway.com/index.php/JHETP/article/view/586. [cited 2019 May 16].

[27] Powell BJ, Bosk EA, Wilen JS, Danko CM, Van Scoyoc A, Banman A, Daro D, Cohn Donnelly A, Huang LA, Powell BJ (2015). Evidence-based programs in “real world” settings: finding the best fit. Advances in child abuse prevention knowledge: the perspective of new leadership [internet].

[28] Forgatch MS, Patterson GR (2010). Cascading effects following intervention. Evid-Based Psychother Child Adolesc.

[29] Forgatch MS, Patterson GR, DeGarmo D, Beldvas ZG (2009). Testing the Oregon delinquency model with 9-year follow-up of the Oregon divorce study. Dev Psychopathol.

[30] Patterson GR, Forgatch MS, DeGarmo DS (2010). Cascading effects following intervention. Dev Psychopathol.

[31] Weisz JR, Kazdin AE. Evidence-based psychotherapies for children and adolescents, Third Edition. Switzerland: Guilford Publications; 2017. p. 641.

[32] Knutson NM, Forgatch MS, Rains LA, Sigmasdottir M, Domenech Rodriguez. Fidelity of implementation rating system (FIMP): the manual for GenerationPMTO. 3rd ed. Eugene: Implementation Sciences International; 2019.

[33] Thijssen J, Albrecht G, Muris P, de Ruiter C (2017). Treatment Fidelity during therapist initial training is related to subsequent effectiveness of parent management training—Oregon model. J Child Fam Stud.

[34] Forgatch MS, DeGarmo DS (2011). Sustaining fidelity following the nationwide PMTO™ implementation in Norway. Prev Sci.

[35] Hukkelberg SS, Ogden T (2013). Working alliance and treatment fidelity as predictors of externalizing problem behaviors in parent management training. J Consult Clin Psychol.

[36] Baumann AA, Domenech Rodriguez MM, Amador Buenabad N, Forgatch MS (2014). Parent management training-Oregon model (PMTO) in Mexico City: integrating cultural adaptation activities in an implementation model. Clin Psychol Sci Pract.

[37] BAUMANN ANA, DOMENECH RODRÍGUEZ MELANIE, PARRA-CARDONA JOSÉ RUBÉN (2011). Community-Based Applied Research With Latino Immigrant Families: Informing Practice and Research According to Ethical and Social Justice Principles. Family Process.

[38] Baumann AA, Mejia A, Lachman JM, Parra-Cardona R, López-Zerón G, Amador Buenabad NG, et al. Parenting programs for underserved populations in low- and middle-income countries: issues of scientific integrity and social justice. Glob Soc Welf. 2018 [cited 2019 Feb 9]; 10.1007/s40609-018-0121-0. 10.1007/s40609-018-0121-0 PMC7036747 32095423

[39] Domenech Rodríguez MM, Baumann AA, Vázquez AL, Amador-Buenabad NG, Franceschi Rivera N, Ortiz-Pons N (2018). Scaling out evidence-based interventions outside the U.S. mainland: social justice or Trojan horse?. J Lat Psychol.

[40] Forgatch MS, Patterson GR, DeGarmo DS (2005). Evaluating fidelity: predictive validity for a measure of competent adherence to the Oregon model of parent management training. Behav Ther.

[41] Sigmarsdóttir M, Forgatch MS, Guðmundsdóttir EV, Thorlacius Ö, Svendsen GT, Tjaden J (2018). Implementing an evidence-based intervention for children in Europe: evaluating the full-transfer approach. J Clin Child Adolesc Psychol.

[42] Forgatch MS, Patterson GR, Gerwitz AH (2013). Looking forward: the promise of widespread implementation of parent training programs. Perspect Psychol Sci.

[43] Proctor EK, Powell BJ, McMillen JC (2013). Implementation strategies: recommendations for specifying and reporting. Implement Sci.

[44] Powell BJ, McMillen JC, Proctor EK, Carpenter CR, Griffey RT, Bunger AC (2012). A compilation of strategies for implementing clinical innovations in health and mental health. Med Care Res Rev.

[45] Aarons GA, Green AE, Willging CE, Ehrhart MG, Roesch SC, Hecht DB (2014). Mixed-method study of a conceptual model of evidence-based intervention sustainment across multiple public-sector service settings. Implement Sci.

[46] Leech NL, Onwuegbuzie AJ (2010). Guidelines for conducting and reporting mixed research in the field of counseling and beyond. J Couns Dev.

[47] Hsieh Li-Yang, Lu Ying-Jui, Lee Yao-Hsien (2014). Using the Technology Acceptance Model to Explore the Behavioral Intentions toward Blended Learning. Communications in Computer and Information Science.

[48] Morgan DL, Krueger RA. Successful Focus Groups: Advancing the State of the Art. In: When to use focus groups and why—SAGE research methods [Internet]. Thousand Oaks; 1993. p. 3–19. Available from: 10.4135/9781483349008.n1. [cited 2019 Feb 20].

[49] Forgatch MS, DeGarmo DS, Beldavs ZG (2005). An efficacious theory-based intervention for stepfamilies. Behav Ther.

[50] Ogden T, Hagen KA (2008). Treatment effectiveness of parent management training in Norway: a randomized controlled trial of children with conduct problems. J Consult Clin Psychol.

[51] Braun V, Clarke V (2006). Using thematic analysis in psychology. Qual Res Psychol.

[52] Gewirtz AH, DeGarmo DS, Zamir O (2018). After deployment, adaptive parenting tools: 1-year outcomes of an evidence-based parenting program for military families following deployment. Prev Sci.

[53] Baumann AA, Rodríguez MMD, Amador NG, Forgatch MS, Parra-Cardona JR (2014). Parent management training-Oregon model (PMTO™) in Mexico city: integrating cultural adaptation activities in an implementation model. Clin Psychol Sci Pract.

[54] Parra‐Cardona Rubén, López‐Zerón Gabriela, Leija Silvia Gisela, Maas Megan K., Villa Monica, Zamudio Efraín, Arredondo Melecia, Yeh Hsueh‐Han, Domenech Rodríguez Melanie M. (2018). A Culturally Adapted Intervention for Mexican‐Origin Parents of Adolescents: The Need to Overtly Address Culture and Discrimination in Evidence‐Based Practice. Family Process.

[55] Fairburn Christopher G, Allen Elizabeth, Bailey-Straebler Suzanne, O'Connor Marianne E, Cooper Zafra (2017). Scaling Up Psychological Treatments: A Countrywide Test of the Online Training of Therapists. Journal of Medical Internet Research.

[56] Cooper Zafra, Bailey-Straebler Suzanne, Morgan Katy E, O'Connor Marianne E, Caddy Caroline, Hamadi Layla, Fairburn Christopher G (2017). Using the Internet to Train Therapists: Randomized Comparison of Two Scalable Methods. Journal of Medical Internet Research.

[57] German RE, Adler A, Frankel SA, Wiltsey Stirman S, Pinedo P, Evans A (2017). Testing a web-based, trained-peer model to build capacity for evidence-based practices in community mental health systems | psychiatric services. Psychiatr Serv.

[58] Klest S (2014). Clustering practitioners within service organizations may improve implementation outcomes for evidence-based programs. Z Für Psychol.

[59] Sheridan SM, Smith TE, Moorman Kim E, Beretvas SN, Park S (2019). A meta-analysis of family-school interventions and children’s social-emotional functioning: moderators and components of efficacy. Rev Educ Res.

[60] Partain PI, Kumbamu A, Asiedu GB, Cristiani V, Deling M, Weis C (2019). Evaluation of community programs for early childhood development: parental perspectives and recommendations. Matern Child Health J.

[61] U.S. Department of Education, Office of Planning, Evaluation, and Policy Development (2009). Evaluation of evidence-based practices in online learning: a meta-analysis and review of online learning studies.

[62] Damschroder LJ, Aron DC, Keith RE, Kirsh SR, Alexander JA, Lowery JC (2009). Fostering implementation of health services research findings into practice: a consolidated framework for advancing implementation science. Implement Sci.

[63] Domenech Rodríguez MM, Baumann AA, Schwartz AL (2011). Cultural adaptation of an evidence based intervention: from theory to practice in a Latino/a community context. Am J Community Psychol.

[64] Ray ML, Wilson MM, Wandersman A, Meyers DC, Katz J (2012). Using a training-of-trainers approach and proactive technical assistance to bring evidence based programs to scale: an operationalization of the interactive systems Framework’s support system. Am J Community Psychol.

[65] Wagner EH, Glasgow RE, Davis C, Bonimi AE, Provost L, McCulloch D (2011). Quality improvement in chronic illness care: a collaborative approach. J Qual Improv.

[66] Ivers NM, Grimshaw JM, Jamtveat G, Flottorp S, O’Brien MA, French SD (2014). Growing literature, stagnant science? Systematic review, meta-regression and cumulative analysis of audit and feedback interventions in health care. J Gen Intern Med.

[67] Brehaut JC, Colquhoun HL, Eva KW, Carroll K, Sales A, Michie S (2016). Practice feedback interventions: 15 suggestions for optimizing EffectivenessPractice feedback interventions. Ann Intern Med.

